# Monoclinic modification of bis­(μ_2_-pyridine-2,6-dicarboxyl­ato)-κ^4^ *O* ^2^,*N*,*O* ^6^:*O* ^6^;κ^4^ *O* ^2^:*O* ^2^,*N*,*O* ^6^-bis­[aqua­dibutyl­tin(IV)]

**DOI:** 10.1107/S1600536811002935

**Published:** 2011-01-29

**Authors:** Seik Weng Ng

**Affiliations:** a Department of Chemistry, University of Malaya, 50603 Kuala Lumpur, Malaysia

## Abstract

The Sn^IV^ atom in the centrosymmetric dinuclear title compound, [Sn_2_(C_4_H_9_)_4_(C_7_H_3_NO_4_)_2_(H_2_O)_2_], exists in a *trans*-C_2_SnNO_4_ penta­gonal–bipyramidal geometry. There are two half-mol­ecules in the asymmetric unit that are completed by inversion symmetry. The crystal studied was a non-merohedral twin with a ratio of 47.3 (1)% for the minor twin component. Bond dimensions are similar to those found in the tetra­gonal polymorph [Huber *et al.* (1989 ▶). *Acta Cryst.* C**45**, 51–54]. O—H⋯O hydrogen-bonding interactions stabilize the crystal packing.

## Related literature

For the tetra­gonal polymorph, see: Huber *et al.* (1989 ▶).
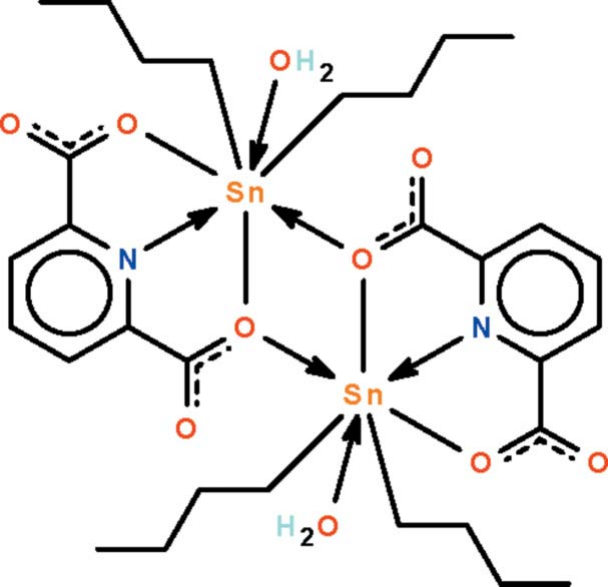


## Experimental

### 

#### Crystal data

[Sn_2_(C_4_H_9_)_4_(C_7_H_3_NO_4_)_2_(H_2_O)_2_]
*M* *_r_* = 832.07
Monoclinic, 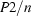

*a* = 16.8882 (8) Å
*b* = 11.0957 (4) Å
*c* = 18.0940 (8) Å
β = 90.251 (4)°
*V* = 3390.5 (3) Å^3^
*Z* = 4
Mo *K*α radiation
μ = 1.53 mm^−1^
*T* = 100 K
0.30 × 0.25 × 0.20 mm

#### Data collection

Agilent SuperNova Dual with Atlas detector diffractometer
Absorption correction: multi-scan (*CrysAlis PRO*; Agilent, 2010 ▶) *T*_min_ = 0.767, *T*_max_ = 1.000
25121 measured reflections
12181 independent reflections
9129 reflections with *I* > 2σ(*I*)
*R*_int_ = 0.054

#### Refinement

*R*[*F* ^2^ > 2σ(*F* ^2^)] = 0.038
*wR*(*F* ^2^) = 0.126
*S* = 1.05
12181 reflections
400 parameters
H-atom parameters constrained
Δρ_max_ = 1.58 e Å^−3^
Δρ_min_ = −1.81 e Å^−3^

### 

Data collection: *CrysAlis PRO* (Agilent, 2010 ▶); cell refinement: *CrysAlis PRO*; data reduction: *CrysAlis PRO*; program(s) used to solve structure: *SHELXS97* (Sheldrick, 2008 ▶); program(s) used to refine structure: *SHELXL97* (Sheldrick, 2008 ▶); molecular graphics: *X-SEED* (Barbour, 2001 ▶); software used to prepare material for publication: *publCIF* (Westrip, 2010 ▶).

## Supplementary Material

Crystal structure: contains datablocks global, I. DOI: 10.1107/S1600536811002935/bt5468sup1.cif

Structure factors: contains datablocks I. DOI: 10.1107/S1600536811002935/bt5468Isup2.hkl

Additional supplementary materials:  crystallographic information; 3D view; checkCIF report

## Figures and Tables

**Table 1:** Hydrogen-bond geometry (Å, °)

*D*—H⋯*A*	*D*—H	H⋯*A*	*D*⋯*A*	*D*—H⋯*A*
O1*w*—H1*w*1⋯O4^i^	0.84	1.81	2.635 (4)	166
O1*w*—H1*w*2⋯O6	0.84	1.98	2.695 (4)	142
O2*w*—H2*w*1⋯O8^ii^	0.84	1.83	2.647 (4)	165
O2*w*—H2*w*2⋯O2^iii^	0.84	1.94	2.719 (4)	153

Symmetry codes: (i) 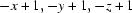; (ii) 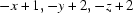; (iii) 
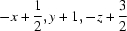
.

**Fig. 1.**
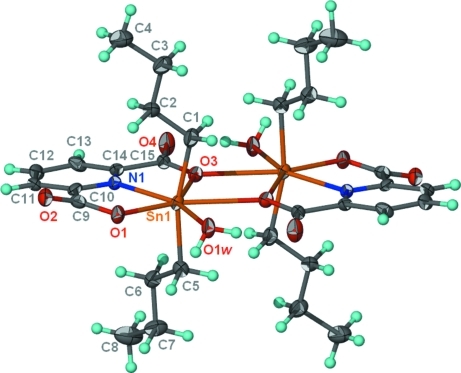
Anisotropic displacement ellipsoid plot (Barbour, 2001) of one of the independent molecules of [Sn(H2O)(C4H9)2(C7H3NO4)]2 at the 70% probability level; hydrogen atoms are drawn as spheres of arbitrary radius.

**Fig. 2.**
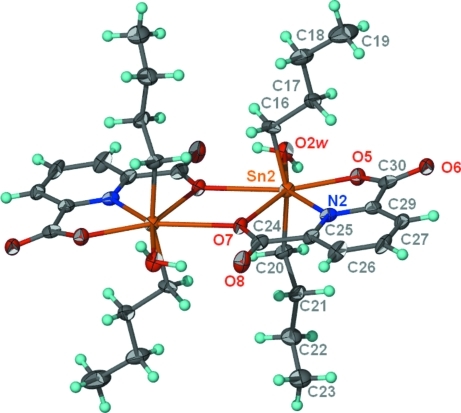
Anisotropic displacement ellipsoid plot (Barbour, 2001) of the second dinuclear molecule.

## supplementary crystallographic information

### Comment

Bis[aquadibutyl(2,6-pyridinedicarboxylato)tin] (Scheme I), which was synthesized by condensing dibutyltin oxide with 2,6-pyridinedicarboxylic acid in methanol, is reported to belong to the tetragonal *P*4_2_/*n* space group [a 17.684 (2), c 11.148 (12) Å; V 3486 (5) Å^3^]. The carboxylate dianion chelates to the tin atom in a tridentate manner; the asymmetric unit is connected to an inversion-related molecule by a long oxygen_carboxyl_–tin bond [2.783 (4) Å] (Huber *et al.*, 1989). A different synthetic route but with the same solvent has yielded the title monoclinic polymorph (Fig. 1). The tin atom shows *trans*-pentagonal bipyramidal coordination with the alkyl groups being in the apical positions [C–Sn–C 166.2 (3) °]. The chelating carboxylate uses one of the two carboxyl oxygen atoms (that which is not involved in chelation) to bind about a center-of-inversion to generate a dinuclear molecule [Sn–O 2.671 (3), 2.689 (3) Å]. The C_2_Sn angles are similar to those of the tetragonal modification. The two independent molecules are linked by extensive O···H···O hydrogen bonds to form a three-dimensional nework.

### Experimental

Di-*n*-butyltin diisothiocyanate (1 mmol) and 2,6-pyridinedicarboxylic acid (1 mmol) were loaded into a convection tube. The tube was filled with dry methanol and kept at 333 K. Colorless crystals were collected from the side arm after several days.

### Refinement

Hydrogen were placed in calculated positions [C—H 0.95 to 0.99 and O–H 0.84 Å; *U*_iso_(H) 1.2 to 1.5*U*_eq_(C,O)] and were included in the refinement in the riding model approximation.

The crystal studied is a non-merohedral twin (twin law: 0.069, 0, 0.931/0, -1, 0/1.069, 0, -0.069) with a ratio of 47.3 (1) % for the minor twin component. The final difference Fourier map had a peak at 0.93 Å from Sn2 and a hole at 1.09 Å from C2. The twin law was given by the *CrysAlis PRO* software.

### Crystal data

**Table:**

[Sn_2_(C_4_H_9_)_4_(C_7_H_3_NO_4_)_2_(H_2_O)_2_]	*F*(000) = 1680
*M**_r_* = 832.07	*D*_x_ = 1.631 Mg m^−^^3^
Monoclinic, *P*2/*n*	Mo *K*α radiation, λ = 0.71073 Å
Hall symbol: -P 2yac	Cell parameters from 11217 reflections
*a* = 16.8882 (8) Å	θ = 2.2–29.4°
*b* = 11.0957 (4) Å	µ = 1.53 mm^−^^1^
*c* = 18.0940 (8) Å	*T* = 100 K
β = 90.251 (4)°	Block, colorless
*V* = 3390.5 (3) Å^3^	0.30 × 0.25 × 0.20 mm
*Z* = 4	

### Data collection

**Table:**

Agilent SuperNova Dual with Atlas detector diffractometer	12181 independent reflections
Radiation source: SuperNova (Mo) X-ray Source	9129 reflections with *I* > 2σ(*I*)
Mirror	*R*_int_ = 0.054
Detector resolution: 10.4041 pixels mm^-1^	θ_max_ = 27.6°, θ_min_ = 2.2°
ω scans	*h* = −21→20
Absorption correction: multi-scan (*CrysAlis PRO*; Agilent, 2010)	*k* = −14→14
*T*_min_ = 0.767, *T*_max_ = 1.000	*l* = −23→23
25121 measured reflections	

### Refinement

**Table:**

Refinement on *F*^2^	Primary atom site location: structure-invariant direct methods
Least-squares matrix: full	Secondary atom site location: difference Fourier map
*R*[*F*^2^ > 2σ(*F*^2^)] = 0.038	Hydrogen site location: inferred from neighbouring sites
*wR*(*F*^2^) = 0.126	H-atom parameters constrained
*S* = 1.05	*w* = 1/[σ^2^(*F*_o_^2^) + (0.0608*P*)^2^] where *P* = (*F*_o_^2^ + 2*F*_c_^2^)/3
12181 reflections	(Δ/σ)_max_ = 0.001
400 parameters	Δρ_max_ = 1.58 e Å^−^^3^
0 restraints	Δρ_min_ = −1.81 e Å^−^^3^

### Fractional atomic coordinates and isotropic or equivalent isotropic displacement parameters (Å^2^)

**Table:**

	*x*	*y*	*z*	*U*_iso_*/*U*_eq_	
Sn1	0.399301 (17)	0.48229 (3)	0.572478 (16)	0.01440 (8)	
Sn2	0.432572 (17)	0.98366 (3)	0.898790 (16)	0.01470 (9)	
O1	0.29816 (18)	0.4421 (3)	0.64381 (16)	0.0175 (7)	
O2	0.21061 (17)	0.3039 (3)	0.67936 (17)	0.0187 (7)	
O3	0.50412 (17)	0.3733 (3)	0.50538 (16)	0.0173 (7)	
O4	0.5499 (2)	0.1918 (3)	0.47472 (18)	0.0279 (8)	
O5	0.36299 (18)	0.9449 (3)	0.79949 (16)	0.0188 (7)	
O6	0.32794 (19)	0.8071 (3)	0.71597 (17)	0.0229 (7)	
O7	0.48802 (16)	0.8760 (3)	1.00654 (15)	0.0159 (6)	
O8	0.50111 (19)	0.6946 (3)	1.06117 (18)	0.0240 (7)	
O1w	0.34736 (18)	0.6700 (3)	0.59441 (17)	0.0210 (7)	
H1w1	0.3778	0.7228	0.5773	0.032*	
H1w2	0.3424	0.6803	0.6402	0.032*	
O2w	0.42201 (18)	1.1692 (3)	0.84214 (17)	0.0193 (7)	
H2w1	0.4476	1.2206	0.8666	0.029*	
H2w2	0.3742	1.1897	0.8400	0.029*	
N1	0.3915 (2)	0.2801 (3)	0.58810 (19)	0.0158 (8)	
N2	0.4107 (2)	0.7816 (3)	0.8935 (2)	0.0159 (8)	
C1	0.3352 (3)	0.4873 (4)	0.4731 (3)	0.0230 (10)	
H1A	0.3172	0.5711	0.4649	0.028*	
H1B	0.3718	0.4668	0.4324	0.028*	
C2	0.2646 (3)	0.4061 (4)	0.4674 (2)	0.0227 (10)	
H2A	0.2306	0.4202	0.5110	0.027*	
H2B	0.2832	0.3216	0.4698	0.027*	
C3	0.2144 (3)	0.4204 (4)	0.3988 (3)	0.0249 (11)	
H3A	0.2475	0.4034	0.3549	0.030*	
H3B	0.1965	0.5051	0.3953	0.030*	
C4	0.1424 (3)	0.3386 (5)	0.3968 (3)	0.0373 (13)	
H4A	0.1129	0.3520	0.3508	0.056*	
H4B	0.1083	0.3566	0.4391	0.056*	
H4C	0.1595	0.2543	0.3994	0.056*	
C5	0.4806 (3)	0.5117 (4)	0.6600 (3)	0.0207 (10)	
H5A	0.5211	0.5697	0.6428	0.025*	
H5B	0.4518	0.5504	0.7012	0.025*	
C6	0.5233 (3)	0.4003 (4)	0.6903 (2)	0.0212 (10)	
H6A	0.5540	0.3626	0.6499	0.025*	
H6B	0.4833	0.3411	0.7069	0.025*	
C7	0.5797 (3)	0.4278 (5)	0.7551 (3)	0.0279 (11)	
H7A	0.6187	0.4893	0.7395	0.033*	
H7B	0.5489	0.4615	0.7968	0.033*	
C8	0.6233 (3)	0.3152 (5)	0.7809 (3)	0.0384 (14)	
H8A	0.6583	0.3358	0.8224	0.058*	
H8B	0.6549	0.2830	0.7402	0.058*	
H8C	0.5849	0.2545	0.7968	0.058*	
C9	0.2753 (3)	0.3336 (4)	0.6549 (2)	0.0173 (9)	
C10	0.3346 (3)	0.2387 (4)	0.6322 (2)	0.0150 (9)	
C11	0.3312 (3)	0.1182 (4)	0.6541 (2)	0.0167 (9)	
H11	0.2906	0.0905	0.6861	0.020*	
C12	0.3885 (3)	0.0404 (4)	0.6278 (2)	0.0201 (10)	
H12	0.3883	−0.0419	0.6423	0.024*	
C13	0.4459 (3)	0.0826 (4)	0.5804 (2)	0.0192 (10)	
H13	0.4850	0.0297	0.5613	0.023*	
C14	0.4457 (3)	0.2041 (4)	0.5609 (2)	0.0154 (9)	
C15	0.5051 (3)	0.2612 (4)	0.5090 (2)	0.0189 (10)	
C16	0.5466 (3)	0.9732 (4)	0.8504 (2)	0.0183 (9)	
H16A	0.5673	1.0559	0.8436	0.022*	
H16B	0.5826	0.9307	0.8849	0.022*	
C17	0.5478 (3)	0.9083 (4)	0.7758 (2)	0.0233 (10)	
H17A	0.5240	0.8274	0.7819	0.028*	
H17B	0.5145	0.9537	0.7403	0.028*	
C18	0.6303 (3)	0.8940 (5)	0.7435 (3)	0.0299 (12)	
H18A	0.6651	0.8547	0.7805	0.036*	
H18B	0.6525	0.9747	0.7328	0.036*	
C19	0.6300 (4)	0.8194 (5)	0.6728 (3)	0.0496 (18)	
H19A	0.6842	0.8120	0.6542	0.074*	
H19B	0.5968	0.8592	0.6355	0.074*	
H19C	0.6087	0.7390	0.6831	0.074*	
C20	0.3346 (3)	1.0267 (4)	0.9664 (3)	0.0200 (10)	
H20A	0.3525	1.0849	1.0044	0.024*	
H20B	0.2945	1.0680	0.9354	0.024*	
C21	0.2945 (3)	0.9221 (4)	1.0054 (3)	0.0207 (10)	
H21A	0.3344	0.8776	1.0348	0.025*	
H21B	0.2726	0.8662	0.9679	0.025*	
C22	0.2278 (3)	0.9627 (5)	1.0564 (3)	0.0277 (11)	
H22A	0.2483	1.0250	1.0907	0.033*	
H22B	0.1850	0.9996	1.0264	0.033*	
C23	0.1939 (3)	0.8590 (5)	1.1008 (3)	0.0349 (13)	
H23A	0.1504	0.8884	1.1316	0.052*	
H23B	0.2355	0.8248	1.1325	0.052*	
H23C	0.1741	0.7967	1.0671	0.052*	
C24	0.4781 (3)	0.7627 (4)	1.0112 (3)	0.0187 (9)	
C25	0.4334 (2)	0.7055 (4)	0.9464 (2)	0.0167 (9)	
C26	0.4195 (3)	0.5834 (4)	0.9416 (3)	0.0232 (10)	
H26	0.4369	0.5303	0.9796	0.028*	
C27	0.3793 (3)	0.5397 (4)	0.8800 (3)	0.0248 (11)	
H27	0.3684	0.4560	0.8755	0.030*	
C28	0.3552 (3)	0.6188 (4)	0.8250 (3)	0.0206 (10)	
H28	0.3281	0.5906	0.7823	0.025*	
C29	0.3718 (3)	0.7404 (4)	0.8340 (2)	0.0183 (9)	
C30	0.3515 (3)	0.8379 (4)	0.7786 (3)	0.0194 (9)	

### Atomic displacement parameters (Å^2^)

**Table:**

	*U*^11^	*U*^22^	*U*^33^	*U*^12^	*U*^13^	*U*^23^
Sn1	0.01628 (16)	0.00938 (14)	0.01755 (16)	−0.00094 (11)	0.00007 (12)	0.00030 (11)
Sn2	0.01571 (16)	0.01010 (15)	0.01830 (17)	0.00106 (11)	−0.00022 (12)	0.00099 (11)
O1	0.0229 (17)	0.0106 (14)	0.0191 (16)	0.0012 (12)	0.0024 (13)	0.0019 (12)
O2	0.0172 (16)	0.0179 (16)	0.0208 (17)	−0.0013 (13)	−0.0003 (13)	0.0046 (13)
O3	0.0208 (16)	0.0107 (15)	0.0205 (16)	−0.0007 (12)	−0.0010 (13)	−0.0006 (12)
O4	0.039 (2)	0.0124 (16)	0.032 (2)	−0.0010 (14)	0.0171 (17)	−0.0029 (14)
O5	0.0219 (17)	0.0168 (15)	0.0175 (16)	−0.0018 (13)	−0.0019 (13)	0.0019 (13)
O6	0.0253 (19)	0.0262 (18)	0.0172 (17)	−0.0086 (14)	−0.0010 (14)	−0.0001 (14)
O7	0.0160 (16)	0.0123 (15)	0.0195 (16)	−0.0022 (12)	−0.0021 (13)	−0.0018 (12)
O8	0.0295 (19)	0.0124 (15)	0.0299 (19)	−0.0023 (13)	−0.0089 (15)	0.0049 (13)
O1w	0.0275 (18)	0.0133 (15)	0.0224 (17)	−0.0016 (13)	0.0083 (14)	0.0008 (13)
O2w	0.0202 (17)	0.0111 (14)	0.0267 (18)	0.0017 (12)	−0.0045 (14)	0.0018 (13)
N1	0.019 (2)	0.0130 (17)	0.0154 (19)	−0.0037 (14)	−0.0050 (15)	0.0033 (15)
N2	0.0150 (19)	0.0149 (18)	0.0177 (19)	0.0002 (14)	0.0038 (15)	0.0020 (15)
C1	0.023 (3)	0.020 (2)	0.026 (3)	−0.0022 (19)	−0.002 (2)	0.0073 (19)
C2	0.032 (3)	0.023 (2)	0.014 (2)	−0.003 (2)	0.001 (2)	−0.0015 (18)
C3	0.025 (3)	0.020 (2)	0.029 (3)	−0.003 (2)	−0.008 (2)	−0.001 (2)
C4	0.039 (3)	0.048 (3)	0.025 (3)	−0.012 (3)	−0.007 (2)	−0.005 (2)
C5	0.021 (2)	0.024 (2)	0.018 (2)	−0.0018 (19)	0.0028 (18)	−0.0009 (18)
C6	0.023 (3)	0.020 (2)	0.020 (2)	−0.0039 (19)	−0.0019 (19)	0.0002 (18)
C7	0.030 (3)	0.028 (3)	0.025 (3)	0.003 (2)	−0.009 (2)	−0.010 (2)
C8	0.048 (4)	0.036 (3)	0.032 (3)	0.007 (3)	−0.011 (3)	−0.009 (2)
C9	0.020 (2)	0.016 (2)	0.015 (2)	−0.0026 (18)	−0.0008 (18)	−0.0010 (17)
C10	0.018 (2)	0.014 (2)	0.013 (2)	−0.0029 (17)	−0.0051 (17)	−0.0015 (16)
C11	0.022 (2)	0.017 (2)	0.011 (2)	−0.0041 (17)	−0.0005 (18)	−0.0001 (17)
C12	0.035 (3)	0.008 (2)	0.017 (2)	0.0010 (18)	−0.0030 (19)	0.0022 (17)
C13	0.030 (3)	0.013 (2)	0.014 (2)	0.0043 (19)	−0.0029 (19)	0.0027 (17)
C14	0.025 (2)	0.012 (2)	0.010 (2)	−0.0004 (17)	−0.0020 (18)	−0.0026 (16)
C15	0.027 (3)	0.017 (2)	0.012 (2)	−0.0039 (18)	0.0005 (19)	−0.0010 (17)
C16	0.015 (2)	0.022 (2)	0.018 (2)	0.0042 (18)	0.0051 (18)	0.0000 (18)
C17	0.025 (3)	0.027 (2)	0.018 (2)	−0.007 (2)	0.005 (2)	−0.0028 (19)
C18	0.024 (3)	0.027 (3)	0.038 (3)	−0.005 (2)	0.012 (2)	−0.003 (2)
C19	0.047 (4)	0.042 (3)	0.060 (4)	−0.012 (3)	0.034 (3)	−0.022 (3)
C20	0.019 (2)	0.019 (2)	0.022 (2)	−0.0018 (18)	0.0036 (19)	0.0000 (18)
C21	0.023 (2)	0.015 (2)	0.025 (3)	−0.0070 (18)	0.003 (2)	−0.0005 (18)
C22	0.024 (3)	0.038 (3)	0.021 (2)	0.003 (2)	0.003 (2)	−0.004 (2)
C23	0.026 (3)	0.044 (3)	0.035 (3)	−0.001 (2)	0.009 (2)	−0.006 (3)
C24	0.013 (2)	0.016 (2)	0.026 (3)	0.0012 (17)	0.0009 (18)	0.0023 (18)
C25	0.013 (2)	0.015 (2)	0.022 (2)	0.0008 (17)	0.0007 (18)	0.0039 (18)
C26	0.016 (2)	0.016 (2)	0.037 (3)	−0.0010 (18)	−0.005 (2)	0.003 (2)
C27	0.018 (2)	0.018 (2)	0.038 (3)	−0.0097 (19)	−0.002 (2)	−0.007 (2)
C28	0.014 (2)	0.024 (2)	0.024 (2)	−0.0018 (18)	0.0025 (18)	−0.0076 (19)
C29	0.013 (2)	0.022 (2)	0.021 (2)	−0.0035 (17)	0.0022 (18)	0.0017 (18)
C30	0.013 (2)	0.022 (2)	0.023 (2)	−0.0013 (18)	0.0043 (18)	0.0024 (19)

### Geometric parameters (Å, °)

**Table:**

Sn1—C1	2.095 (5)	C7—C8	1.523 (7)
Sn1—C5	2.116 (5)	C7—H7A	0.9900
Sn1—O1	2.191 (3)	C7—H7B	0.9900
Sn1—N1	2.265 (3)	C8—H8A	0.9800
Sn1—O1w	2.296 (3)	C8—H8B	0.9800
Sn1—O3	2.468 (3)	C8—H8C	0.9800
Sn1—O3^i^	2.689 (3)	C9—C10	1.511 (6)
Sn2—C20	2.116 (4)	C10—C11	1.395 (6)
Sn2—C16	2.122 (4)	C11—C12	1.383 (6)
Sn2—O5	2.185 (3)	C11—H11	0.9500
Sn2—N2	2.274 (4)	C12—C13	1.380 (6)
Sn2—O2w	2.307 (3)	C12—H12	0.9500
Sn2—O7	2.467 (3)	C13—C14	1.393 (6)
Sn2—O7^ii^	2.671 (3)	C13—H13	0.9500
O1—C9	1.281 (5)	C14—C15	1.516 (6)
O2—C9	1.226 (5)	C16—C17	1.529 (6)
O3—C15	1.246 (5)	C16—H16A	0.9900
O4—C15	1.246 (5)	C16—H16B	0.9900
O5—C30	1.261 (5)	C17—C18	1.522 (6)
O6—C30	1.247 (5)	C17—H17A	0.9900
O7—C24	1.272 (5)	C17—H17B	0.9900
O8—C24	1.239 (5)	C18—C19	1.524 (7)
O1w—H1w1	0.8400	C18—H18A	0.9900
O1w—H1w2	0.8400	C18—H18B	0.9900
O2w—H2w1	0.8400	C19—H19A	0.9800
O2w—H2w2	0.8400	C19—H19B	0.9800
N1—C10	1.333 (5)	C19—H19C	0.9800
N1—C14	1.340 (5)	C20—C21	1.519 (6)
N2—C25	1.331 (5)	C20—H20A	0.9900
N2—C29	1.339 (5)	C20—H20B	0.9900
C1—C2	1.498 (6)	C21—C22	1.526 (6)
C1—H1A	0.9900	C21—H21A	0.9900
C1—H1B	0.9900	C21—H21B	0.9900
C2—C3	1.509 (6)	C22—C23	1.518 (7)
C2—H2A	0.9900	C22—H22A	0.9900
C2—H2B	0.9900	C22—H22B	0.9900
C3—C4	1.518 (7)	C23—H23A	0.9800
C3—H3A	0.9900	C23—H23B	0.9800
C3—H3B	0.9900	C23—H23C	0.9800
C4—H4A	0.9800	C24—C25	1.530 (6)
C4—H4B	0.9800	C25—C26	1.378 (6)
C4—H4C	0.9800	C26—C27	1.391 (6)
C5—C6	1.532 (6)	C26—H26	0.9500
C5—H5A	0.9900	C27—C28	1.386 (7)
C5—H5B	0.9900	C27—H27	0.9500
C6—C7	1.538 (6)	C28—C29	1.387 (6)
C6—H6A	0.9900	C28—H28	0.9500
C6—H6B	0.9900	C29—C30	1.514 (6)
			
C1—Sn1—C5	165.64 (17)	C6—C7—H7B	109.3
C1—Sn1—O1	96.31 (15)	H7A—C7—H7B	108.0
C5—Sn1—O1	95.48 (15)	C7—C8—H8A	109.5
C1—Sn1—N1	95.91 (15)	C7—C8—H8B	109.5
C5—Sn1—N1	95.61 (15)	H8A—C8—H8B	109.5
O1—Sn1—N1	71.27 (12)	C7—C8—H8C	109.5
C1—Sn1—O1w	85.88 (15)	H8A—C8—H8C	109.5
C5—Sn1—O1w	88.72 (15)	H8B—C8—H8C	109.5
O1—Sn1—O1w	77.48 (11)	O2—C9—O1	125.4 (4)
N1—Sn1—O1w	148.72 (12)	O2—C9—C10	120.2 (4)
C1—Sn1—O3	87.68 (15)	O1—C9—C10	114.4 (4)
C5—Sn1—O3	88.82 (14)	N1—C10—C11	122.0 (4)
O1—Sn1—O3	138.84 (10)	N1—C10—C9	113.7 (4)
N1—Sn1—O3	67.57 (11)	C11—C10—C9	124.2 (4)
O1w—Sn1—O3	143.64 (11)	C12—C11—C10	118.1 (4)
C1—Sn1—O3^i^	81.19 (14)	C12—C11—H11	121.0
C5—Sn1—O3^i^	84.66 (14)	C10—C11—H11	121.0
O1—Sn1—O3^i^	154.94 (10)	C13—C12—C11	119.8 (4)
N1—Sn1—O3^i^	133.74 (11)	C13—C12—H12	120.1
O1w—Sn1—O3^i^	77.47 (10)	C11—C12—H12	120.1
O3—Sn1—O3^i^	66.18 (11)	C12—C13—C14	119.0 (4)
C20—Sn2—C16	164.57 (17)	C12—C13—H13	120.5
C20—Sn2—O5	95.78 (15)	C14—C13—H13	120.5
C16—Sn2—O5	97.78 (14)	N1—C14—C13	121.1 (4)
C20—Sn2—N2	96.85 (15)	N1—C14—C15	114.9 (4)
C16—Sn2—N2	94.39 (15)	C13—C14—C15	124.0 (4)
O5—Sn2—N2	71.61 (12)	O4—C15—O3	126.8 (4)
C20—Sn2—O2w	89.79 (15)	O4—C15—C14	117.0 (4)
C16—Sn2—O2w	86.22 (14)	O3—C15—C14	116.1 (4)
O5—Sn2—O2w	76.71 (11)	C17—C16—Sn2	113.9 (3)
N2—Sn2—O2w	148.11 (12)	C17—C16—H16A	108.8
C20—Sn2—O7	86.99 (14)	Sn2—C16—H16A	108.8
C16—Sn2—O7	87.57 (14)	C17—C16—H16B	108.8
O5—Sn2—O7	139.04 (10)	Sn2—C16—H16B	108.8
N2—Sn2—O7	67.49 (11)	H16A—C16—H16B	107.7
O2w—Sn2—O7	144.24 (10)	C18—C17—C16	113.9 (4)
C20—Sn2—O7^ii^	83.65 (14)	C18—C17—H17A	108.8
C16—Sn2—O7^ii^	80.95 (13)	C16—C17—H17A	108.8
O5—Sn2—O7^ii^	155.31 (10)	C18—C17—H17B	108.8
N2—Sn2—O7^ii^	133.04 (11)	C16—C17—H17B	108.8
O2w—Sn2—O7^ii^	78.60 (10)	H17A—C17—H17B	107.7
O7—Sn2—O7^ii^	65.65 (11)	C17—C18—C19	112.3 (4)
C9—O1—Sn1	121.3 (3)	C17—C18—H18A	109.1
C15—O3—Sn1	118.3 (3)	C19—C18—H18A	109.1
C30—O5—Sn2	120.9 (3)	C17—C18—H18B	109.1
C24—O7—Sn2	118.8 (3)	C19—C18—H18B	109.1
Sn1—O1w—H1w1	109.5	H18A—C18—H18B	107.9
Sn1—O1w—H1w2	109.5	C18—C19—H19A	109.5
H1w1—O1w—H1w2	109.5	C18—C19—H19B	109.5
Sn2—O2w—H2w1	109.5	H19A—C19—H19B	109.5
Sn2—O2w—H2w2	109.5	C18—C19—H19C	109.5
H2w1—O2w—H2w2	109.5	H19A—C19—H19C	109.5
C10—N1—C14	119.9 (4)	H19B—C19—H19C	109.5
C10—N1—Sn1	117.3 (3)	C21—C20—Sn2	116.6 (3)
C14—N1—Sn1	122.5 (3)	C21—C20—H20A	108.1
C25—N2—C29	119.9 (4)	Sn2—C20—H20A	108.1
C25—N2—Sn2	123.3 (3)	C21—C20—H20B	108.1
C29—N2—Sn2	116.7 (3)	Sn2—C20—H20B	108.1
C2—C1—Sn1	116.8 (3)	H20A—C20—H20B	107.3
C2—C1—H1A	108.1	C20—C21—C22	112.8 (4)
Sn1—C1—H1A	108.1	C20—C21—H21A	109.0
C2—C1—H1B	108.1	C22—C21—H21A	109.0
Sn1—C1—H1B	108.1	C20—C21—H21B	109.0
H1A—C1—H1B	107.3	C22—C21—H21B	109.0
C1—C2—C3	116.0 (4)	H21A—C21—H21B	107.8
C1—C2—H2A	108.3	C23—C22—C21	112.2 (4)
C3—C2—H2A	108.3	C23—C22—H22A	109.2
C1—C2—H2B	108.3	C21—C22—H22A	109.2
C3—C2—H2B	108.3	C23—C22—H22B	109.2
H2A—C2—H2B	107.4	C21—C22—H22B	109.2
C2—C3—C4	113.8 (4)	H22A—C22—H22B	107.9
C2—C3—H3A	108.8	C22—C23—H23A	109.5
C4—C3—H3A	108.8	C22—C23—H23B	109.5
C2—C3—H3B	108.8	H23A—C23—H23B	109.5
C4—C3—H3B	108.8	C22—C23—H23C	109.5
H3A—C3—H3B	107.7	H23A—C23—H23C	109.5
C3—C4—H4A	109.5	H23B—C23—H23C	109.5
C3—C4—H4B	109.5	O8—C24—O7	127.6 (4)
H4A—C4—H4B	109.5	O8—C24—C25	117.3 (4)
C3—C4—H4C	109.5	O7—C24—C25	115.1 (4)
H4A—C4—H4C	109.5	N2—C25—C26	122.1 (4)
H4B—C4—H4C	109.5	N2—C25—C24	115.3 (4)
C6—C5—Sn1	116.5 (3)	C26—C25—C24	122.6 (4)
C6—C5—H5A	108.2	C25—C26—C27	118.4 (4)
Sn1—C5—H5A	108.2	C25—C26—H26	120.8
C6—C5—H5B	108.2	C27—C26—H26	120.8
Sn1—C5—H5B	108.2	C28—C27—C26	119.7 (4)
H5A—C5—H5B	107.3	C28—C27—H27	120.2
C5—C6—C7	113.7 (4)	C26—C27—H27	120.2
C5—C6—H6A	108.8	C27—C28—C29	118.3 (4)
C7—C6—H6A	108.8	C27—C28—H28	120.9
C5—C6—H6B	108.8	C29—C28—H28	120.9
C7—C6—H6B	108.8	N2—C29—C28	121.7 (4)
H6A—C6—H6B	107.7	N2—C29—C30	113.4 (4)
C8—C7—C6	111.6 (4)	C28—C29—C30	124.9 (4)
C8—C7—H7A	109.3	O6—C30—O5	125.3 (4)
C6—C7—H7A	109.3	O6—C30—C29	118.5 (4)
C8—C7—H7B	109.3	O5—C30—C29	116.1 (4)
			
C1—Sn1—O1—C9	86.4 (3)	C14—N1—C10—C11	2.9 (6)
C5—Sn1—O1—C9	−101.8 (3)	Sn1—N1—C10—C11	−170.7 (3)
N1—Sn1—O1—C9	−7.7 (3)	C14—N1—C10—C9	−176.7 (4)
O1w—Sn1—O1—C9	170.7 (3)	Sn1—N1—C10—C9	9.8 (5)
O3—Sn1—O1—C9	−7.3 (4)	O2—C9—C10—N1	162.0 (4)
O3^i^—Sn1—O1—C9	169.1 (3)	O1—C9—C10—N1	−15.9 (5)
C1—Sn1—O3—C15	−96.4 (3)	O2—C9—C10—C11	−17.6 (7)
C5—Sn1—O3—C15	97.6 (3)	O1—C9—C10—C11	164.5 (4)
O1—Sn1—O3—C15	0.6 (4)	N1—C10—C11—C12	−0.9 (6)
N1—Sn1—O3—C15	1.0 (3)	C9—C10—C11—C12	178.6 (4)
O1w—Sn1—O3—C15	−176.2 (3)	C10—C11—C12—C13	−1.0 (6)
O3^i^—Sn1—O3—C15	−177.8 (4)	C11—C12—C13—C14	1.1 (7)
C20—Sn2—O5—C30	−104.5 (3)	C10—N1—C14—C13	−2.8 (6)
C16—Sn2—O5—C30	82.8 (3)	Sn1—N1—C14—C13	170.4 (3)
N2—Sn2—O5—C30	−9.2 (3)	C10—N1—C14—C15	177.6 (4)
O2w—Sn2—O5—C30	167.1 (3)	Sn1—N1—C14—C15	−9.1 (5)
O7—Sn2—O5—C30	−12.4 (4)	C12—C13—C14—N1	0.9 (7)
O7^ii^—Sn2—O5—C30	168.1 (3)	C12—C13—C14—C15	−179.6 (4)
C20—Sn2—O7—C24	97.0 (3)	Sn1—O3—C15—O4	174.6 (4)
C16—Sn2—O7—C24	−97.4 (3)	Sn1—O3—C15—C14	−5.7 (5)
O5—Sn2—O7—C24	1.6 (4)	N1—C14—C15—O4	−170.7 (4)
N2—Sn2—O7—C24	−1.7 (3)	C13—C14—C15—O4	9.8 (7)
O2w—Sn2—O7—C24	−177.6 (3)	N1—C14—C15—O3	9.5 (6)
O7^ii^—Sn2—O7—C24	−178.6 (4)	C13—C14—C15—O3	−170.0 (4)
C1—Sn1—N1—C10	−96.8 (3)	C20—Sn2—C16—C17	−168.9 (5)
C5—Sn1—N1—C10	91.8 (3)	O5—Sn2—C16—C17	−17.5 (3)
O1—Sn1—N1—C10	−2.1 (3)	N2—Sn2—C16—C17	54.5 (3)
O1w—Sn1—N1—C10	−5.0 (4)	O2w—Sn2—C16—C17	−93.6 (3)
O3—Sn1—N1—C10	178.2 (3)	O7—Sn2—C16—C17	121.7 (3)
O3^i^—Sn1—N1—C10	179.7 (2)	O7^ii^—Sn2—C16—C17	−172.6 (3)
C1—Sn1—N1—C14	89.8 (3)	Sn2—C16—C17—C18	−176.4 (3)
C5—Sn1—N1—C14	−81.6 (3)	C16—C17—C18—C19	175.0 (4)
O1—Sn1—N1—C14	−175.5 (3)	C16—Sn2—C20—C21	−121.9 (6)
O1w—Sn1—N1—C14	−178.4 (3)	O5—Sn2—C20—C21	86.6 (3)
O3—Sn1—N1—C14	4.8 (3)	N2—Sn2—C20—C21	14.5 (3)
O3^i^—Sn1—N1—C14	6.3 (4)	O2w—Sn2—C20—C21	163.2 (3)
C20—Sn2—N2—C25	−82.1 (4)	O7—Sn2—C20—C21	−52.4 (3)
C16—Sn2—N2—C25	87.3 (3)	O7^ii^—Sn2—C20—C21	−118.2 (3)
O5—Sn2—N2—C25	−176.0 (4)	Sn2—C20—C21—C22	176.5 (3)
O2w—Sn2—N2—C25	177.2 (3)	C20—C21—C22—C23	−174.1 (4)
O7—Sn2—N2—C25	1.7 (3)	Sn2—O7—C24—O8	−179.4 (4)
O7^ii^—Sn2—N2—C25	5.6 (4)	Sn2—O7—C24—C25	1.5 (5)
C20—Sn2—N2—C29	96.9 (3)	C29—N2—C25—C26	1.2 (7)
C16—Sn2—N2—C29	−93.7 (3)	Sn2—N2—C25—C26	−179.9 (3)
O5—Sn2—N2—C29	3.1 (3)	C29—N2—C25—C24	179.3 (4)
O2w—Sn2—N2—C29	−3.8 (4)	Sn2—N2—C25—C24	−1.7 (5)
O7—Sn2—N2—C29	−179.2 (3)	O8—C24—C25—N2	−179.2 (4)
O7^ii^—Sn2—N2—C29	−175.4 (3)	O7—C24—C25—N2	0.0 (6)
C5—Sn1—C1—C2	−176.9 (6)	O8—C24—C25—C26	−1.0 (7)
O1—Sn1—C1—C2	−31.8 (4)	O7—C24—C25—C26	178.2 (4)
N1—Sn1—C1—C2	39.9 (4)	N2—C25—C26—C27	−1.0 (7)
O1w—Sn1—C1—C2	−108.7 (4)	C24—C25—C26—C27	−179.0 (4)
O3—Sn1—C1—C2	107.1 (4)	C25—C26—C27—C28	0.7 (7)
O3^i^—Sn1—C1—C2	173.4 (4)	C26—C27—C28—C29	−0.5 (7)
Sn1—C1—C2—C3	173.8 (3)	C25—N2—C29—C28	−1.0 (6)
C1—C2—C3—C4	−178.3 (4)	Sn2—N2—C29—C28	180.0 (3)
C1—Sn1—C5—C6	−128.1 (7)	C25—N2—C29—C30	−179.0 (4)
O1—Sn1—C5—C6	86.7 (3)	Sn2—N2—C29—C30	2.0 (5)
N1—Sn1—C5—C6	15.1 (3)	C27—C28—C29—N2	0.6 (7)
O1w—Sn1—C5—C6	164.0 (3)	C27—C28—C29—C30	178.4 (4)
O3—Sn1—C5—C6	−52.3 (3)	Sn2—O5—C30—O6	−163.8 (3)
O3^i^—Sn1—C5—C6	−118.4 (3)	Sn2—O5—C30—C29	13.3 (5)
Sn1—C5—C6—C7	−178.4 (3)	N2—C29—C30—O6	167.6 (4)
C5—C6—C7—C8	−177.4 (4)	C28—C29—C30—O6	−10.3 (7)
Sn1—O1—C9—O2	−162.7 (3)	N2—C29—C30—O5	−9.7 (6)
Sn1—O1—C9—C10	15.1 (5)	C28—C29—C30—O5	172.4 (4)

Symmetry codes: (i) −*x*+1, −*y*+1, −*z*+1; (ii) −*x*+1, −*y*+2, −*z*+2.

### Hydrogen-bond geometry (Å, °)

**Table:**

*D*—H···*A*	*D*—H	H···*A*	*D*···*A*	*D*—H···*A*
O1w—H1w1···O4^i^	0.84	1.81	2.635 (4)	166
O1w—H1w2···O6	0.84	1.98	2.695 (4)	142
O2w—H2w1···O8^ii^	0.84	1.83	2.647 (4)	165
O2w—H2w2···O2^iii^	0.84	1.94	2.719 (4)	153

Symmetry codes: (i) −*x*+1, −*y*+1, −*z*+1; (ii) −*x*+1, −*y*+2, −*z*+2; (iii) −*x*+1/2, *y*+1, −*z*+3/2.
